# Biosorption of B-aflatoxins Using Biomasses Obtained from Formosa Firethorn [*Pyracantha koidzumii* (Hayata) Rehder]

**DOI:** 10.3390/toxins8070218

**Published:** 2016-07-13

**Authors:** Rosa Adriana Ramales-Valderrama, Alma Vázquez-Durán, Abraham Méndez-Albores

**Affiliations:** 1 BUAP, Department of Food Engineering, Faculty of Chemical Engineering, Puebla 72570, Mexico; rosa_ramales@outlook.com; 2 UNAM–FESC, Campus 4. Multidisciplinary Research Unit L14 (Food, Mycotoxins and Mycotoxicosis), Cuautitlan Izcalli 54714, Mexico; vazquezd.alma@yahoo.com.mx

**Keywords:** B-aflatoxins, *Pyracantha koidzumii*, biomaterials, sorption

## Abstract

Mycotoxin adsorption onto biomaterials is considered as a promising alternative for decontamination without harmful chemicals. In this research, the adsorption of B-aflatoxins (AFB_1_ and AFB_2_) using *Pyracantha koidzumii* biomasses (leaves, berries and the mixture of leaves/berries) from aqueous solutions was explored. The biosorbent was used at 0.5% (*w*/*v*) in samples spiked with 100 ng/mL of B-aflatoxin standards and incubated at 40 °C for up to 24 h. A standard biosorption methodology was employed and aflatoxins were quantified by an immunoaffinity column and UPLC methodologies. The biosorbent-aflatoxin interaction mechanism was investigated from a combination of zeta potential (ζ), Fourier transform infrared spectroscopy (FTIR) and scanning electron microscopy (SEM). The highest aflatoxin uptakes were 86% and 82% at 6 h using leaves and the mixture of leaves/berries biomasses, respectively. A moderate biosorption of 46% was attained when using berries biomass. From kinetic studies, the biosorption process is described using the first order adsorption model. Evidence from FTIR spectra suggests the participation of hydroxyl, amine, carboxyl, amide, phosphate and ketone groups in the biosorption and the mechanism was proposed to be dominated by the electrostatic interaction between the negatively charged functional groups and the positively charged aflatoxin molecules. Biosorption by *P. koidzumii* biomasses has been demonstrated to be an alternative to conventional systems for B-aflatoxins removal.

## 1. Introduction

The term “*mycotoxin*” refers to a large number of closely related toxic secondary metabolites produced by fungi growing on different agricultural commodities. Among more than 300 mycotoxins described so far, aflatoxins (AF) are the toxins of major health concern. AF are produced mainly by strains of *Aspergillus flavus* Link, *A. parasiticus* Speare and *A. nomius* Kurtzman et al. [1,2]. Four principal AF are produced by those fungi: aflatoxin B_1_ (AFB_1_), aflatoxin B_2_ (AFB_2_), aflatoxin G_1_ (AFG_1_), and aflatoxin G_2_ (AFG_2_). AFB_1_, the most commonly encountered toxic metabolite in this group causes severe liver damage and has higher toxicity and carcinogenicity than other AF; therefore, it has been classified as a human carcinogen [3].

The ingestion of AF-contaminated food or feed causes intoxications known as aflatoxicosis. In animal husbandry, aflatoxicosis impairs animal health, welfare and productivity causing severe economic losses [4]. Moreover, accumulation of AF in animal tissues may result in an indirect exposure to humans by consuming the contaminated products, as demonstrated for AFB_1_ and its metabolite aflatoxin M_1_ (AFM_1_) [5]. Under these circumstances, the most threatening aspect of contamination is related to the presence of AF in food of animal origin such as meat, milk or eggs [6,7].

The Food and Agriculture Organization (FAO) estimates that at least 25% of world cereal production is contaminated with mycotoxins [8]. For this reason, a number of methods have been investigated in connection with their effectiveness to control AF in contaminated feedstuffs [9]; the aims of these methods are either to inactivate, degrade or to remove the toxin, and can be classified into biological, chemical and physical [10]. Biological methods have not been transferred into practice, these protocols mainly include procedures with microorganisms. One example is the conversion of AFB_1_—principally by *Flavobacterium aurantiacum*—to harmless degradation by-products. However, the conversions are generally slow and incomplete [11]. AF can also be degraded chemically; nevertheless, most of the chemical processes that have been investigated are impractical, unsafe and compromise the nutritional, sensory and functional properties of the treated commodity. However, our research group recently reported a secure, effective, inexpensive and environmentally friendly method to detoxify AF-contaminated maize using neutral electrolyzed oxidizing water [12]. Finally, physical methods are focused on AF removal using different adsorbents added to contaminated diets in order to be effective in the gastro-intestinal tract [13]. At present, however, the utilization of binding adsorbents is the most applied technique for protecting animals against AF harmful effects. Consequently, the development of an efficient, inexpensive and environmentally friendly method to remove AF either completely or up to acceptable levels—while retaining the nutritional value of the treated commodity—is an attractive alternative.

In recent times, biosorption technology has emerged as a promising option over conventional binding technologies with the advantages of low-cost, greater profitability, ease of operation and greater efficiency [14]. Biosorption can be defined as the uptake of contaminants via various physicochemical mechanisms including ion-exchange, adsorption, complexation, chelation and micro-precipitation by biological materials [15]. Some studies have demonstrated that different kinds of plant biomasses interact with molecules and remove hazardous materials from solutions [16,17,18]. Previously, it has been demonstrated that the biomass obtained from *Pyracantha coccinea* berries is capable of removing the cationic methylene blue dye [19,20,21]. However, there is currently a lack of information regarding the use of *Pyracantha koidzumii* biomasses as a biosorbent for B-aflatoxins removal. *Pyracantha koidzumii* (Hayata) Rehder is a thorny evergreen shrub native to Taiwan province of China. This one and several closely related species (*Rosaceae* family) are widely cultivated in Mexico for ornamental and hedges purposes since the 18th century. Despite the use of this plant in traditional medicine due to its multiple health benefits, there is limited information about other potential applications. Therefore, the present research was conducted to evaluate the biosorption potential of *P. koidzumii* biomasses in an attempt to propose a novel, natural, abundant, inexpensive and effective binder for B-aflatoxins adsorption from aqueous solutions.

## 2. Results and Discussion

### 2.1. Biosorption of B-aflatoxins

**Figure 1:**
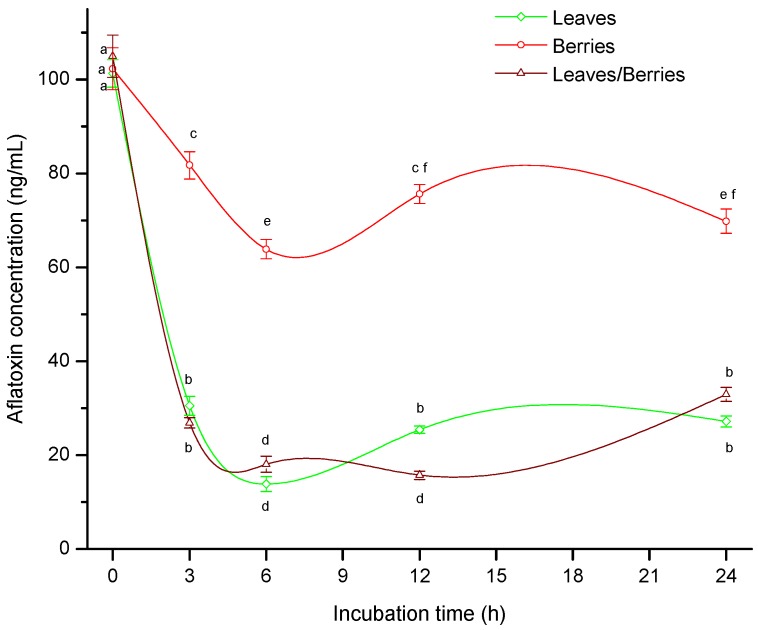
Biosorption of B-aflatoxins at 40 °C onto *P. koidzumii* biomasses (leaves, berries and the mixture of leaves/berries) as a function of incubation time. Data were expressed as mean values ± standard error of three independent experiments.

Results of the biosorption studies are shown in Figure 1. In general, biosorption capacity of the three different biomasses increased with increasing incubation time and the maximum level occurred at 6 h; subsequently, AF uptake capacity did not change significantly up to 24 h. At 6 h incubation, leaves alone and the mixture of leaves/berries showed the highest efficiency against AF removal, biosorption percentages were 86% and 82%, respectively. In these samples, the final AF concentrations were 13.87 and 18.07 ng/mL, respectively. In both cases, the AF concentrations were below the recommended maximum limit of 20 ng/g that Mexico considers for contamination in maize destined for human consumption [22]. On the contrary, at 6 h incubation, berries biomass showed a moderate AF uptake capacity, reaching values up to 46% (Figure 1). During the biosorption assay (at 6 h incubation) the three different *P. koidzumii* biomasses presented notable differences in AF uptake capacity as seen in the UPLC chromatograms (Figure S1).

In this regard, various adsorbents such as activated charcoal, zeolite, hydrated sodium calcium aluminosilicates (HSCAS), montmorillonite, smectite, polymers and also yeast and products from yeast have been tested for this purpose [23]. It has been demonstrated that one sequestering agent based on a yeast cell wall product (MTB-100) with an inclusion rate of 0.05%, significantly reduced AFM_1_ contamination of milk (59%) in late-lactation Holstein cows fed a diet containing 100 ng/g total AF (55 ng/g AFB_1_) [24]. In another study, the same product at 0.56% to the diet was not effective in reducing milk AFM_1_ concentrations (4%) in cows consuming 112 ng of AFB_1_/g diet [25]. Although adsorbents reduce the AF content, their practical application has several limitations, since most of them have the disadvantage of showing high reduction of bioavailability of some nutrients. In view of these considerations—besides its excellent nutritional value—the biosorption capacity of *P. koidzumii* biomasses (leaves and the mixture of leaves/berries) would hold greater promise for the development of practical and economical decontamination procedures. 

**Figure 2:**
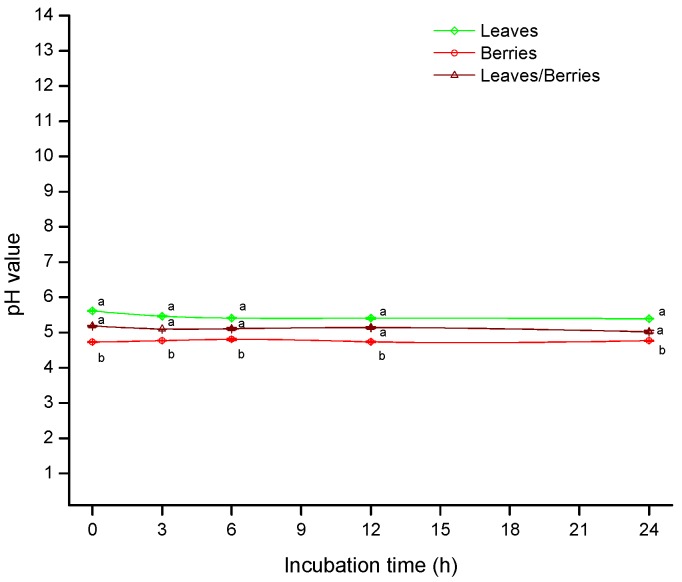
pH values of *P. koidzumii* biomasses (leaves, berries and the mixture of leaves/berries) as a function of incubation time. Data were expressed as mean values ± standard error of three independent experiments.

On the other hand, pH values are shown in Figure 2. At 6 h incubation, leaves and the mixture of leaves/berries presented average pH values of 5.41 and 5.10, respectively. Significant differences were not detected among these treatments. However, a pH value of 4.81 was observed in the berries biomass. As a result, the lower biosorption of AF obtained at low pH values (as in the case of berries biomass) may be explained by the competition of excess oxonium ions (H_3_O^+^) with AF molecules for active biosorption sites of the different functional groups on the biomass surface [26].

### 2.2. Zeta Potential (ζ)

**Figure 3:**
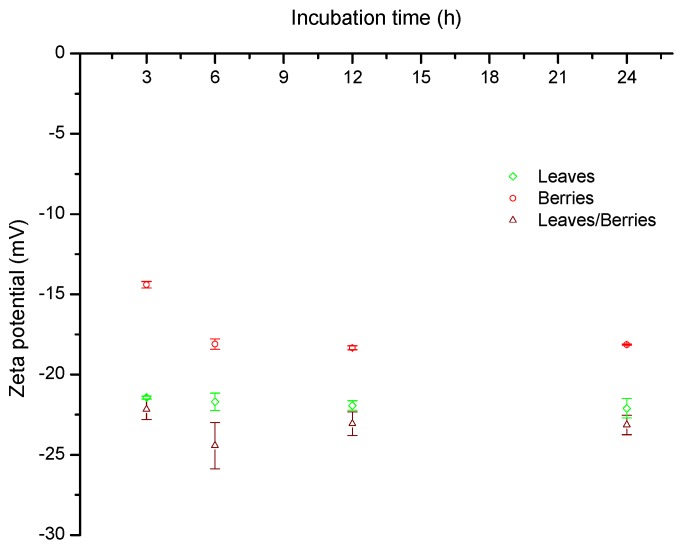
Zeta potential values of *P. koidzumii* biomasses (leaves, berries and the mixture of leaves/berries) as a function of incubation time. Data were expressed as mean values ± standard error of three independent experiments.

Zeta or electrokinetic potential of suspended particles can be defined as the difference in potential between the bulk of the conducting medium and the stationary layer of fluid surrounding the suspended particle [27]. Zeta potential is closely related to the surface charge of colloidal particles. Due to the external charge of particles, when they are exposed to an external electrical field, they will migrate towards an electrode of the opposite charge at a certain velocity called electrophoretic mobility. In this research, the zeta potential increased slightly with increasing incubation time and reached the maximum value at 6 h; subsequently, zeta potential did not change significantly up to 24 h (Figure 3). A zeta potential value of −17.2 mV was observed in berries biomass; however, for leaves and the mixture of leaves/berries biomasses the values were −21.8 mV and −23.2 mV, respectively. These results are in accordance with Akar et al. [19] who reported a zeta potential value of −15.60 mV for *P. coccinea* berries biomass at pH 4. The high negative-charged surface on these biosorbents (leaves and the mixture of leaves/berries) result in a high biosorption uptake because of the enhancement of attractive forces between AF and the biomass surface. Thus, it may be concluded that the interaction type between AF and the biosorbent would be primarily electrostatic in nature, since AF are very polar molecules which possess a substantially high positive charge.

### 2.3. The Influence of Biomass on Aflatoxin Biosorption

The graphic of AF biosorption with time (up to 6 h) was fitted using the following formula, where Co is the initial AF concentration and C is the concentration at time t.

(1) ln(CCo)=−kt

**Figure 4:**
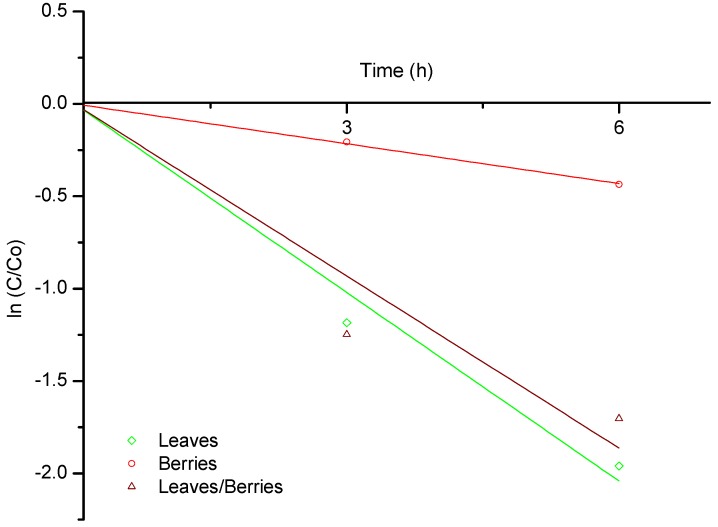
Biosorption rates of B-aflatoxins at 40 °C up to 6 h onto *P. koidzumii* biomasses (leaves, berries and the mixture of leaves/berries).

The graphic provides evidence for first order adsorption kinetics. The estimated biosorption rates at 40 °C for AF in the presence of the three different biomasses are presented in Figure 4. During the assay, the rate of biosorption of AF was higher using leaves and the mixture of leaves/berries biomasses than using berries biomass. Moreover, the biosorption rate constants (k) of AF in the presence of the biomasses were significantly different (Table 1). At 3 h incubation, the biosorption rate constant (k) was higher for the leaves/berries mixture (0.0069), followed by leaves (0.0066). However, at 6 h incubation, the biosorption rate constant (k) was higher for the leaves biomass (0.0054), which is in close agreement with the information presented in Figure 1.

### 2.4. FTIR Analysis

**Figure 5:**
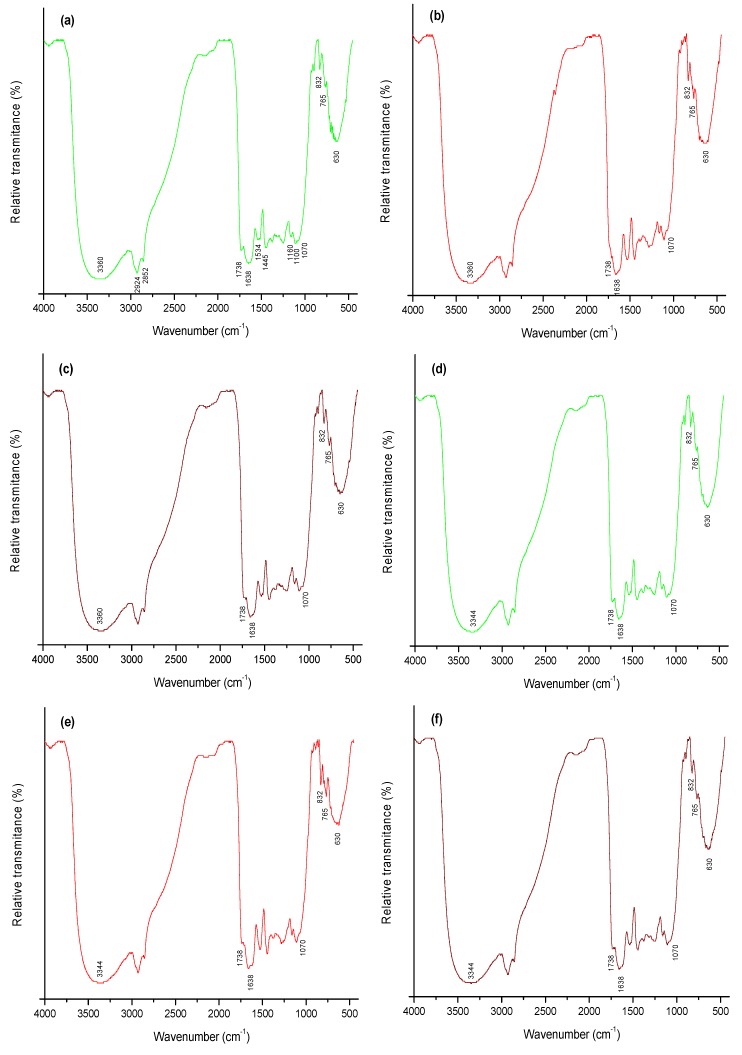
Fourier Transform Infrared Spectroscopy (FTIR) spectrum of *P. koidzumii* biomasses before biosorption: (**a**) leaves; (**b**) berries; (**c**) the mixture of leaves/berries and after 100 ng/mL aflatoxin biosorption: (**d**) leaves; (**e**) berries; (**f**) the mixture of leaves/berries.

FTIR spectra of the three different biomasses before and after AF interaction are shown in Figure 5. In general, spectra before AF interaction (profiles a, b and c) showed a strong and broad absorption band at 3360 cm^−1^, indicative of the presence of –OH groups and –NH groups [28]. The spectra has also alkyl chains (CH*_n_*) at 2924 cm^−1^ and 2852 cm^−1^, respectively [29]. The absorption at 1738 cm^−1^ is the characteristic band of –C=O groups and the –C=O chelate stretching of amide I band was observed at 1638 cm^−1^ [19]. NH_3_ deformation in amino acids appeared at 1534 cm^−1^ and the band in 1445 cm^−1^ can be associated with the in-plane –OH bending vibration in carboxylic acids. Moreover, the bands at 1160 cm^−1^ and 1100 cm^−1^ can be assigned to the –C=S stretching vibration in thiocarbonyl compounds [30]. The band located at 1070 cm^−1^ was attributed to the organic phosphate groups and the bands in 832 cm^−1^ and 765 cm^−1^ are characteristic of –CH out-of plane deformation in substituted aromatic hydrocarbons. Finally, the absorption band at 630 cm^−1^ is attributable to the C–CO–C bend in ketones [30]. The principal components of plant materials are proteins, amino acids, amides, amines, nitrites, nitrates, carbohydrates (starch, sugar, cellulose, hemicelluloses and lignin), lipids and many more compounds such as phytochemicals (terpenoids, phenolics and alkaloids). These components have many different functional groups, which are responsible for the biosorption processes. In this context, the functional groups on the biosorbent surface could be responsible for the biosorption of B-aflatoxins.

The FTIR spectra of AF loaded biomasses are also shown in Figure 5 (profiles d, e and f). In general, comparing the native biomass with that found in AF-loaded one can reveal the following:

- The band at 3360 cm^−1^ shifted slightly to 3344 cm^−1^ after interaction with AF. This frequency shift may be attributed to the interaction between AF and both the hydroxyl and amine groups on the biomass surface. A shift in the spectral frequency is related to an energy change of the functional group and this is indicative that the bonding pattern of the different functional groups changed after AF biosorption [19].
- There was a markedly reduction in intensity of the carboxyl band located at 1738 cm^−1^ and of the –C=O chelate stretching of amide I band at 1638 cm^−1^, confirming the involvement of these functional groups in AF binding onto biomasses.
- There was not a strong shift in the organic phosphate group located at 1070 cm^−1^. However, the phosphate band intensity was significantly reduced after interaction with AF.
- Other significant changes were in the CH out-of plane deformation bands in substituted aromatic hydrocarbons located at 832 cm^−1^ and 765 cm^−1^, respectively. In addition, the absorption band at 630 cm^−1^ attributable to the C–CO–C bend in ketones significantly reduced the intensity after interaction with AF.

In summary, all changes observed in the spectral frequency (wavenumber) and intensity (relative transmittance) of AF-loaded biomasses lead to the conclusion that hydroxyl, amine, carboxyl, amide, phosphate and ketone groups are likely responsible for binding AF molecules. These results agree with those of Akar et al. [19] who reported that the biomass obtained from *P. coccinea* berries is capable of removing the cationic methylene blue dye from aqueous solutions. To the best of our knowledge, the present study is the first one to demonstrate that conventional biomasses from *P. koidzumii* have good potential for the biosorption of AF from aqueous solutions being a natural, abundant, cost-effective and environmentally friendly biomaterial.

### 2.5. Surface Morphology of P. koidzumii Biomasses

**Figure 6:**
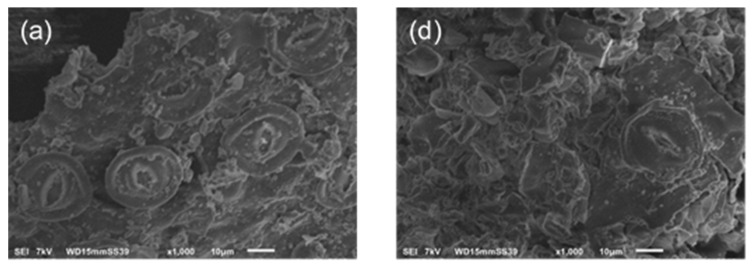

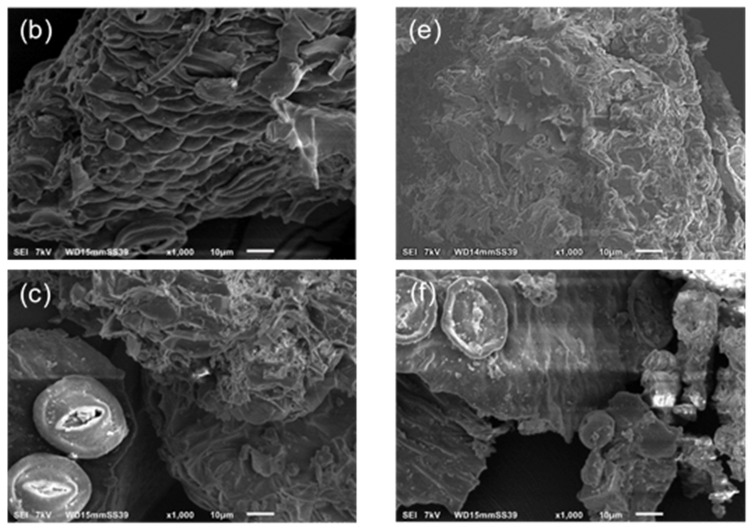
SEM micrographs of *P. koidzumii* biomasses before biosorption: (**a**) leaves; (**b**) berries; (**c**) the mixture of leaves/berries and after 100 ng/mL aflatoxin biosorption: (**d**) leaves; (**e**) berries; (**f**) the mixture of leaves/berries.

The surface morphology and microstructure of biomasses was evaluated using SEM images. Micrographs of the three different biomasses before and after AF interaction are shown in Figure 6. In general, images before AF interaction (profiles a, b and c) showed irregularly dispersed granules and cavities. Leaves and the mixture of leaves/berries biomasses (profiles a and c) presented stomata which had elliptical form and remained open all the time. The stomatal type is cyclocytic, which also has been established for *Cydonia oblonga* Mill [31] and *Amelanchier ovalis Medic* [32], plants belonging to the family *Rosaceae*. However, the difference in the surface morphology after AF uptake by the three different biomasses is evident in Figure 6 (profiles d, e and f); the surface morphology of the biomaterial became rough, stomata remained closed (profiles d and f) and particle agglomeration occurred. Agglomeration is a process that allows materials to gather into a mass or cluster and can either increase or decrease the porosity or density of the material. In this research, all the above produces benefit on the biomasses including increased efficiency and functionality resulting in biomaterials with improved interaction with AF.

## 3. Conclusions

A novel method to remove AF from aqueous solutions using biomasses from *P. koidzumii* has been described. Being of natural origin, it may be safe for use in agricultural commodities. However, biosorbents exhibiting high binding capacities in vitro need to be further tested in livestock to demonstrate its suitability to alleviate the toxic effects of AF, research in this direction is in progress.

## 4. Materials and Methods

### 4.1. Plant Material and Preparation of Biomasses

*P. koidzumii*, cultivated in the Botanic Garden of the Superior Studies Faculty at Cuautitlan (National Autonomous University of Mexico) was collected during September–October 2015. Plant authentication was carried out by the biologists Abel Bonfil and Silvestre Benitez (responsibles of the botanic garden) and a voucher of specimen (reference number: 10479) was deposited in the herbarium of the Department of Biologic Sciences. Plant parts (leaves and berries) used in the experiments were washed with distilled water to remove loosely adhering particles and water-soluble impurities. After cleaning, these anatomical parts were separately dried in a forced air oven at 40 °C, milled in an electric plate-style mill type C-11-1 (Glen Mills Inc., Clifton, NJ, USA) and sieved (60 mesh) to provide ground material with a particle size of <250 µm. The biomasses obtained were stored in vacuum-sealed plastic containers at −20 °C until further analysis.

### 4.2. Aqueous Solution of Aflatoxins

AFB_1_ (312.3 g/mol) and AFB_2_ (314.3 g/mol) obtained from Sigma-Aldrich Co (St. Louis, MO, USA) were dissolved in dimethyl sulfoxide (DMSO) and diluted with distilled water to the desired concentration. The ratio of AFB_1_ to AFB_2_ was 7:3 and was chosen considering that AFB_1_ is the most abundant of the AF family and usually accounts for 70%–95% of the total toxin produced by the fungus *A. flavus* Link [33].

### 4.3. Biosorption Assay

A standard biosorption methodology was applied to evaluate the biomass efficiency using 0.5% (*w*/*v*), the safe limit that the Panel on Additives and Products or Substances used in Animal Feed (FEEDAP) considers for bentonite (a dioctahedral montmorillonite authorized for the reduction of feed contamination by mycotoxins) [34]. A sample of 0.25 g dry weight of each biomass (leaves, berries and the mixture of leaves/berries in a 7:3 ratio) was dispersed in 50 mL of AF solution (100 ng/mL) and incubated in an agitated water bath (Bellco Glass Inc., Vineland, NJ, USA) at 40 °C for 3, 6, 12 and 24 h. At the end of the incubation periods, samples were rapidly cooled and the AF content was determined using the immunoaffinity column (IAC) and UPLC procedures. The pH was immediately determined using a pH meter, Model PC45 (Conductronic, Puebla, Mexico). All determinations were performed in triplicate.

### 4.4. Aflatoxin Analysis

#### 4.4.1. Using Immunoaffinity Columns (IAC)

AF concentration was determined according to the 991.31 AOAC method [35] using antibody-based IAC for AFB_1_ and AFB_2_ (VICAM, Milford, MA, USA). Briefly, the preparation was filtered through a micro-fiber filter, and 10 mL were passed through the IAC (Afla B, VICAM Science Technology, Watertown, MA, USA). After that, the column was washed twice with 10 mL of distilled water and dried with sterile air flow. The toxins were then eluted with 1 mL of HPLC grade methanol and quantified in a fluorometer VICAM Series-4EX (VICAM Source Scientific. Irvine, CA, USA) after reacting with 1 mL of 0.002% aqueous bromine. The detection limit for AF via fluorescence measurement is approximately 0.5 ng/mL.

#### 4.4.2. Using Ultra Performance Liquid Chromatography (UPLC)

AF identification was carried out according to the technique proposed by Jardon-Xicontencatl et al. [12] using a Waters ACQUITY Ultra Performance Liquid Chromatography (UPLC) H-Class System equipped with a quaternary solvent manager and an ACQUITY UPLC BEH C18 phase reverse column (2.1 × 100 mm, 1.7 μm). Standards, as well as samples collected from the IAC (1 μL) were injected and eluted with a single ternary mixture of 64:18:18 water/methanol/acetonitrile (all HPLC grade) at a flow rate of 400 μL/min. AF were fluorometrically detected and identified using an UPLC-optimized fluorescence detector (Waters, Milford, MA, USA). The excitation and emission wavelengths were 365 and 429 nm, respectively. AF were identified by their retention time (Rt) and compared with those for a pure AF standard solution under identical conditions. The estimated detection limits are 0.58 and 2.01 ng/L for AFB_2_ and AFB_1_, respectively.

### 4.5. Characterization of the Biosorbent

#### 4.5.1. Zeta Potential (*ζ*)

Measurement of zeta potential was performed using the ZETASIZER Nano Series ZSP (Malvern Instruments, Worcestershire, UK). Unless stated otherwise, all measurements were carried out at room temperature by diluting 100 µL of the suspension in 2 mL of deionized water. Triplicates of each sample were measured and each measurement comprised 10 runs, in order to find a stable reading. In all cases, the pH of the sample was the original value of the suspension.

#### 4.5.2. Fourier Transform Infrared Spectroscopy (FTIR) Analysis

The functional groups present in the biosorbent before and after interaction with AF were characterized using a FTIR Frontier SP8000 spectrophotometer (Perkin Elmer, Waltham, MA, USA) equipped with a deuterated triglycine sulphate (DTGS) detector and controlled with the software Spectrum 10.4.2. Briefly, the ground samples (<250 µm) were placed in a diffuse reflectance (DR) sample holder and DR spectra were collected in the range of 400–4000 cm^−1^ at a resolution of 4 cm^−1^ by co-adding 32 scans. A background spectrum was obtained against air every day during the experiment. The spectra of both unloaded and AF-loaded biomasses were collected in transmittance mode in quadruplicate and the average value was used.

#### 4.5.3. Scanning Electron Microscopy (SEM)

The surface morphology and microstructure of the biosorbent before and after AF sorption was examined using an in touch scope SEM (JEOL, JSM-6012LA, Tokio, Japan). Samples were coated with a thin layer of gold by a vacuum electric sputter coater (Denton Vacuum Inc., Desk V HP, Moorestown, NJ, USA) operated at 7 mA during 5 min to enhance the electron conductivity and image quality. Microscopy analysis was performed at 1000× with an accelerating voltage of 7 kV under high vacuum.

### 4.6. Experimental Design and Statistical Analysis

The experiment was conducted as a completely randomized 3 × 5 factorial design, fifteen experimental conditions were carried out with three replicates. The first factor corresponds to the biomass type (leaves, berries and the mixture of leaves/berries) and the second one to the incubation periods (0, 3, 6, 12 and 24 h). Data were assessed by two-way analysis of variance (ANOVA) and means comparisons were performed according to the Tukey’s test using the Statistical Analysis System [36]. A significance value of *p* < 0.05 was used to distinguish significant differences between treatments.

## Figures and Tables

**Table 1:** Biosorption rate constants (k (min^−1^)) for B-aflatoxins at 40 °C up to 6 h onto *P. koidzumii* biomasses (leaves, berries and the mixture of leaves/berries).

Incubation Time (h)	Leaves	Berries	Leaves/Berries
0	0	0	0
3	0.0066	0.0011	0.0069
6	0.0054	0.0010	0.0047

## Supplementary Materials

The following are available online at www.mdpi.com/2072-6651/8/7/218/s1, Figure S1: Representative UPLC profiles of biosorption using *Pyracantha koidzumii* biomasses at 6 h incubation. (a) samples spiked with 100 ng/mL of B-aflatoxin standards; (b) samples after the biosorption with leaves biomass; (c) samples after the biosorption with berries biomass; (d) samples after the biosorption with the mixture of leaves/berries biomass. The Rt values for AFB_2_ and AFB_1_ were 3.09 and 3.89 min, respectively.

## Abbreviations

The following abbreviations are used in this manuscript:

UPLC	Ultra performance liquid chromatography
FTIR	Fourier transform infrared spectroscopy
SEM	Scanning electron microscopy
AF	Aflatoxin(s)
AFB_1_	Aflatoxin B_1_
AFB_2_	Aflatoxin B_2_
AFG_1_	Aflatoxin G_1_
AFG_2_	Aflatoxin G_2_
AFM_1_	Aflatoxin M_1_
FAO	Food and agriculture organization
HSCAS	Hydrated sodium calcium aluminosilicates
DMSO	Dimethyl sulfoxide
FEEDAP	Panel on Additives and Products or Substances used in Animal Feed
IAC	Immunoaffinity columns
Rt	Retention time
DTGS	Deuterated triglycine sulphate
DR	Diffuse reflectance
ANOVA	Analysis of variance

## References

[1] Feibelman T.P., Cotty P.J., Doster M.A., Michailides T.J. (1998). A morphologically distinct strain of Aspergillus nomius. Mycologia.

[2] Nesci A., Gsponer N., Etcheverry M. (2007). Natural maize phenolic acids for control of aflatoxigenic fungi on maize. J. Food Sci..

[3] IARC Some Naturally Occurring Substances: Food Items and Constituents, Heterocyclic Aromatic Amines and Mycotoxins. http://bases.bireme.br/cgi-bin/wxislind.exe/iah/online/?IsisScript=iah/iah.xis&src=google&base=WHOLIS&lang=p&nextAction=lnk&exprSearch=9283212568&indexSearch=ID.

[4] Mellor S. (2001). Mycotoxins in feed-a global challenge. Feed Mix.

[5] Trucksess M.W., Richard J.L., Stoloff L., McDonald J.S., Brumley W.C. (1983). Absorption and distribution patterns of aflatoxicol and aflatoxins B1 and M1 in blood and milk of cows given aflatoxin B1. Am. J. Vet. Res..

[6] Herzallah S.M. (2009). Determination of aflatoxins in eggs, milk, meat and meat products using HPLC fluorescent and UV detectors. Food Chem..

[7] Hussain Z., Khan M.Z., Khan A., Javed I., Saleemi M.K., Mahmood S., Asi M.R. (2010). Residues of aflatoxin B1 in broiler meat: Effect of age and dietary aflatoxin B1 levels. Food Chem. Toxicol..

[8] Dowling T.S. (1997). Fumonisin and its toxic effects. Cereal Foods World.

[9] Di Stefano V., Pitonzo R., Cicero N., D’Oca M.C. (2014). Mycotoxin contamination of animal feedingstuff: Detoxification by gamma-irradiation and reduction of aflatoxins and ochratoxin A concentrations. Food Addit. Contam. A.

[10] Doyle M., Applebaum R., Brackett R., Marth E. (1982). Physical, chemical and biological degradation of mycotoxins in foods and agricultural commodities. J. Food Prot..

[11] Bata A., Lasztity R. (1999). Detoxification of mycotoxin-contaminated food and feed by microorganisms. Trends Food Sci. Technol..

[12] Jardon-Xicotencatl S., Diaz-Torres R., Marroquin-Cardona A., Villarreal-Barajas T., Mendez-Albores A. (2015). Detoxification of Aflatoxin-Contaminated Maize by Neutral Electrolyzed Oxidizing Water. Toxins (Basel).

[13] Ramos A.J., FinkGremmels J., Hernandez E. (1996). Prevention of toxic effects of mycotoxins by means of nonnutritive adsorbent compounds. J. Food Prot..

[14] Tunali S., Ozcan A., Kaynak Z., Ozcan A.S., Akar T. (2007). Utilization of the Phaseolus vulgaris L. Waste biomass for decolorization of the textile dye Acid Red 57: Determination of equilibrium, kinetic and thermodynamic parameters. J. Environ. Sci. Health A Toxic/Hazard. Subst. Environ. Eng..

[15] Volesky B. (1994). Advances in biosorption of metals: Selection of biomass types. FEMS Microbiol. Rev..

[16] Schneider I.A.H., Rubio J., Smith R.W. (2001). Biosorption of metals onto plant biomass: Exchange adsorption or surface precipitation?. Int. J. Miner. Process..

[17] Sekhar K.C., Kamala C.T., Chary N.S., Anjaneyulu Y. (2003). Removal of heavy metals using a plant biomass with reference to environmental control. Int. J. Miner. Process..

[18] Sekhar K.C., Kamala C.T., Chary N.S., Sastry A.R.K., Rao T.N., Vairamani M. (2004). Removal of lead from aqueous solutions using an immobilized biomaterial derived from a plant biomass. J. Hazard. Mater..

[19] Akar T., Anilan B., Gorgulu A., Akar S.T. (2009). Assessment of cationic dye biosorption characteristics of untreated and non-conventional biomass: Pyracantha coccinea berries. J. Hazard. Mater..

[20] Akar T., Celik S., Akar S.T. (2010). Biosorption performance of surface modified biomass obtained from Pyracantha coccinea for the decolorization of dye contaminated solutions. Chem. Eng. J..

[21] Ari A.G., Celik S. (2013). Biosorption potential of Orange G dye by modified Pyracantha coccinea: Batch and dynamic flow system applications. Chem. Eng. J..

[22] De la Federación D.O. (2002). Norma Oficial Mexicana NOM-188-SSA1–2002, Productos y Servicios: Control de aflatoxinas en cereales para consumo humano y animal, especificaciones sanitarias. D. Of. Fed..

[23] Huwig A., Freimund S., Kappeli O., Dutler H. (2001). Mycotoxin detoxication of animal feed by different adsorbents. Toxicol. Lett..

[24] Diaz D.E., Hagler W.M., Blackwelder J.T., Eve J.A., Hopkins B.A., Anderson K.L., Jones F.T., Whitlow L.W. (2004). Aflatoxin binders II: Reduction of aflatoxin M1 in milk by sequestering agents of cows consuming aflatoxin in feed. Mycopathologia.

[25] Kutz R.E., Sampson J.D., Pompeu L.B., Ledoux D.R., Spain J.N., Vazquez-Anon M., Rottinghaus G.E. (2009). Efficacy of Solis, NovasilPlus, and MTB-100 to reduce aflatoxin M1 levels in milk of early to mid lactation dairy cows fed aflatoxin B1. J. Dairy Sci..

[26] Batzias F.A., Sidiras D.K. (2007). Simulation of methylene blue adsorption by salts-treated beech sawdust in batch and fixed-bed systems. J. Hazard. Mater..

[27] Delgado A.V., Gonzalez-Caballero E., Hunter R.J., Koopal L.K., Lyklema J. (2005). Measurement and interpretation of electrokinetic phenomena (IUPAC technical report). Pure Appl. Chem..

[28] Park D., Yun Y.S., Park J.M. (2005). Studies on hexavalent chromium biosorption by chemically-treated biomass of Ecklonia sp. Chemosphere.

[29] Yun Y.S., Park D., Park J.M., Volesky B. (2001). Biosorption of trivalent chromium on the brown seaweed biomass. Environ. Sci. Technol..

[30] Shurvell H. (2002). Spectra–Structure Correlations in the Mid-and Far-Infrared. Handbook of Vibrational Spectroscopy.

[31] Ganeva T. (2009). Leaf Epidermis Structure in Cydonia Oblonga Mill.(Rosaceae). Biotechnol. Biotechnol. Equip..

[32] Ganeva T., Uzunova K. (2010). Leaf epidermis structure in Amelanchier ovalis Medic. (Rosaceae). Biotechnol. Biotechnol. Equip..

[33] Mendez-Albores A., Martinez-Bustos F., Gaytan-Martinez M., Moreno-Martinez E. (2008). Effect of lactic and citric acid on the stability of B-aflatoxins in extrusion-cooked sorghum. Lett. Appl. Microbiol..

[34] EFSA (2011). Scientific Opinion on the Safety and Efficacy of Bentonite (dioctahedral montmorillonite) as Feed Additive for all Species. EFSA J..

[35] Horwuitz W. (2000). Official Methods of Analysis of AOAC International.

[36] SAS/STAT User’s Guide. Version 8.

