# Supplementary material for: Biosorption of B-aflatoxins Using Biomasses Obtained from Formosa Firethorn [Pyracantha koidzumii (Hayata) Rehder]
Source: Toxins (Basel). 2016 Jul 13;8(7):218. doi: 10.3390/toxins8070218 (PMC4963850; doi:10.3390/toxins8070218)
Supplement: Supplementary file 1 [file toxins-08-00218-s001.pdf]

Supplementary Materials: Biosorption of B-aflatoxins Using Biomasses Obtained from Formosa Firethorn [*Pyracantha koidzumii* (Hayata) Rehder]

Rosa Adriana Ramales-Valderrama, Alma Vázquez-Durán and Abraham Méndez-Albores

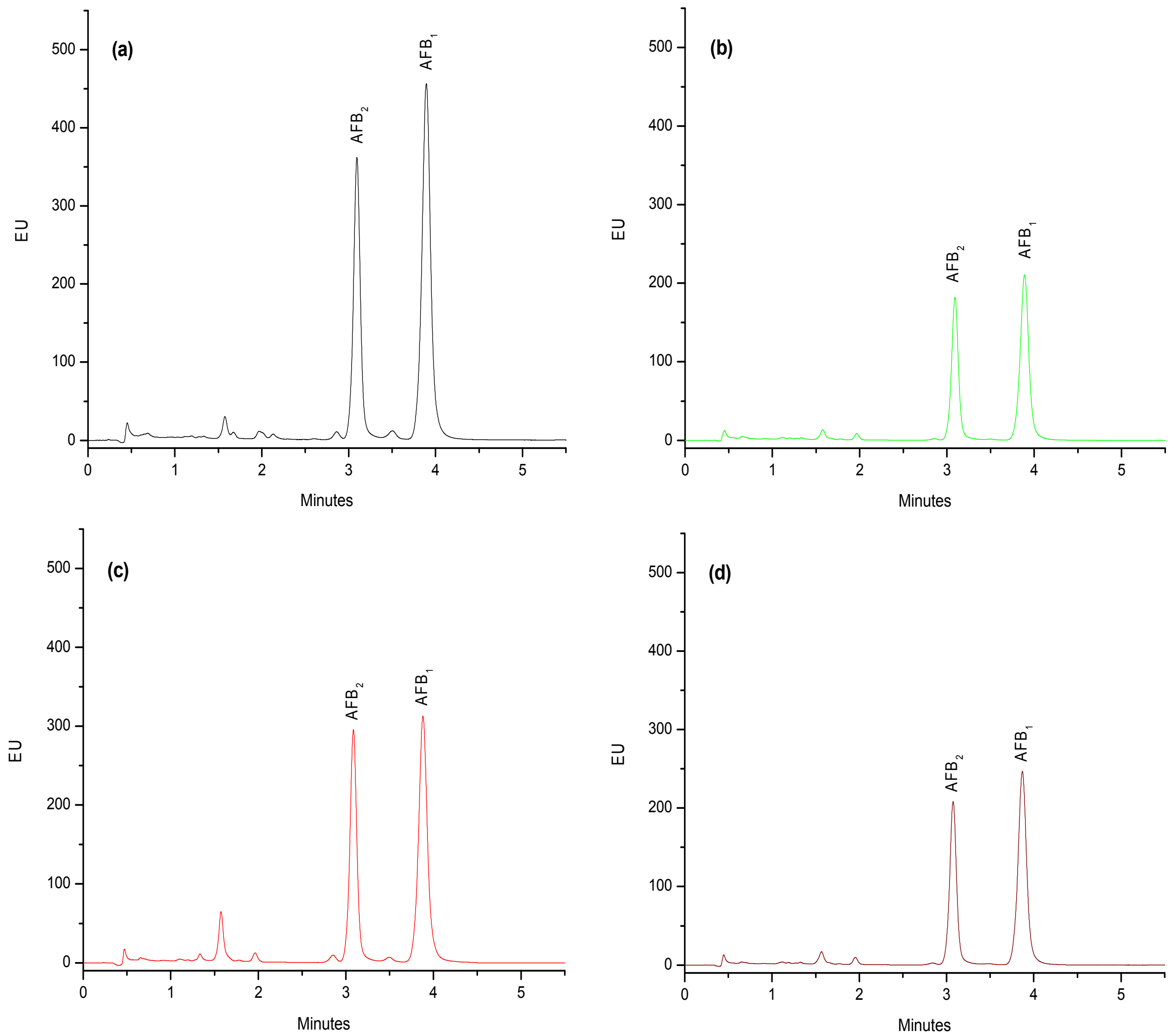

Figure S1. Representative UPLC profiles of biosorption using *P. koidzumii* biomasses at 6 h incubation. (**a**) samples spiked with 100 ng/mL of B-aflatoxin standards; (**b**) samples after the biosorption with leaves biomass; (**c**) samples after the biosorption with berries biomass; (**d**) samples after the biosorption with the mixture of leaves/berries biomass. The Rt values for AFB_2 and AFB_1 were 3.09 and 3.89 min, respectively.
